# Ibogalogs induce antiseizure activity in rodents by a mechanism involving 5-HT_2A/2C_ receptor activation with a major role of 5-HT_2A_ receptors in the hippocampal CA3 subfield

**DOI:** 10.1016/j.bcp.2026.117799

**Published:** 2026-02-12

**Authors:** Abdeslam Chagraoui, Paulina Kazmierska-Grebowska, Olivia Byache, Alejandro Abraham, Deborah Rudin, Bartosz Caban, Tomasz Kowalczyk, Matthias E. Liechti, Daniel Wacker, Chloé Aman, Philippe De Deurwaerdère, Hugo R. Arias

**Affiliations:** a Laboratory Nordic: Neuroendocrine, endocrine and germinal differentiation communication - UMR 1239 (Inserm), Normandy University, UNIROUEN, Institute for Research and Innovation in Biomedicine of Normandy (IRIB), Rouen, France; b Department of Medical Biochemistry, Rouen University Hospital, CHU de Rouen, France; c Department of Neurobiology, Faculty of Biology and Environmental Protection, University of Lodz, Lodz, Poland; d Centre National de la Recherche Scientifique, Institut des Neurosciences Intégratives et Cognitives d’Aquitaine, UMR 5287, Bordeaux, France; e Department of Pharmacological Sciences and Department of Neuroscience, Icahn School of Medicine at Mount Sinai, New York, NY, USA; f Division of Clinical Pharmacology and Toxicology, Department of Pharmaceutical Sciences, University of Basel, Basel, Switzerland; g Division of Clinical Pharmacology and Toxicology, Department of Biomedicine, University Hospital Basel and University of Basel, Basel, Switzerland; h Department of Pharmacology and Physiology, Oklahoma State University College of Osteopathic Medicine, Tahlequah, OK, USA

**Keywords:** Ibogalogs, Antiseizure activity, 5-HT_2A/2C_ receptors, Kainic acid, Monoamines

## Abstract

The antiseizure properties of ibogalogs, including ibogaminalog (DM506), ibogainalog (IBG), and nor-IBG, were assessed in rodents using the pentylenetetrazol (PTZ)-induced seizure test. The behavioral findings indicated that ibogalogs exhibited mild acute antiseizure effects in mice, with endpoint- and time window-dependent differences between the compounds. The antiseizure effect was suppressed by volinanserin and SB242084, consistent with the involvement of 5-HT_2A_ and 5-HT_2C_ receptors. The antiseizure activity after repeated administration (7 and 14 days) of subthreshold doses of nor-IBG (3 mg/kg) or DM506 (5 mg/kg) was higher than that after acute treatment, indicating augmented efficacy. Subthreshold doses of DM506 and nor-IBG restored the impact of PTZ on monoamine levels in hippocampal tissue following repeated administration, but not after a single dose. Additionally, the influence of ibogalogs was evaluated on epileptiform discharges induced by kainic acid (KA) in the CA3 region of the hippocampus. The results showed that nor-IBG and DM506 decreased epileptiform discharges in a concentration-dependent manner. Nor-IBG activity was inhibited by volinanserin, supporting a role for the 5-HT_2A_R. Functional studies have shown that ibogalogs are more potent agonists at 5-HT_2A/2C_Rs than at 5-HT_1A/1B_Rs, supporting the role of 5-HT_2A_R. In conclusion, repetitive treatment with ibogalogs induced antiseizure activity in mice through 5-HT_2A/2C_R activation, accompanied by normalization of PTZ-induced alterations in hippocampal monoamines. In the hippocampal CA3 subfield, ibogalogs reduced KA-induced epileptiform discharges, where nor-IBG activity was mediated by 5-HT_2A_R activation.

## 1. Introduction

Epilepsy is considered a spectrum disorder with symptoms that vary depending on the underlying mechanisms, including more than 150 known gene mutations, excessive excitatory neurotransmission, and various acquired factors [1,2]. The numerous etiologies preclude the development of “one size fits it all” type of medications. Although many therapeutic agents have been approved for the treatment of epilepsy, none cure the disease or modify the epileptogenesis [3]. Currently, pharmacological treatments are applied according to the specific type of epilepsy. Many antiepileptic medications used in clinics today are based on their ability to increase GABAergic (inhibitory) transmission, inhibit glutamatergic (excitatory) transmission, and block Ca^2+^ and Na^+^ channels, which otherwise enhance glutamate (Glu) release and neuronal firing [3–5]. Although several serotonergic drugs are under investigation, fenfluramine is the only approved medication (for severe childhood-onset epilepsies) whose mechanism of action involves the activation of several serotonin (5-HT) receptors, ultimately increasing GABAergic transmission and positively modulating the opioid sigma-1 receptor, which decreases glutamatergic transmission [3,5]. These pharmacodynamic effects correlate with the general view that increased glutamatergic transmission induces overstimulation of the central nervous system (CNS), which may result in seizures [6], as well as with evidence that GABAergic dysfunction can trigger epilepsy and status epilepticus in rodents [7,8] and is closely related to the development of epilepsy in humans [9]. From these mechanisms of action, we can infer that any drug that decreases excitatory transmission and/or increases inhibitory transmission may have potential utility in treating epileptic seizures. Numerous studies on different seizure types in rodents and humans have shown antiseizure activity for selective agonists of the 5-HT_1A/1D_R and 5-HT_2A/2C_R subtypes and selective antagonists of the 5-HT_6_R and 5-HT_7_R subtypes [2,5,10,11]. These results were corroborated by studies involving selective antagonists and knockout (KO) mice lacking the 5-HT_1A_R, 5-HT_2A_R, 5-HT_2C_R, or 5-HT_7_R [5,12].

**Fig. 1.**
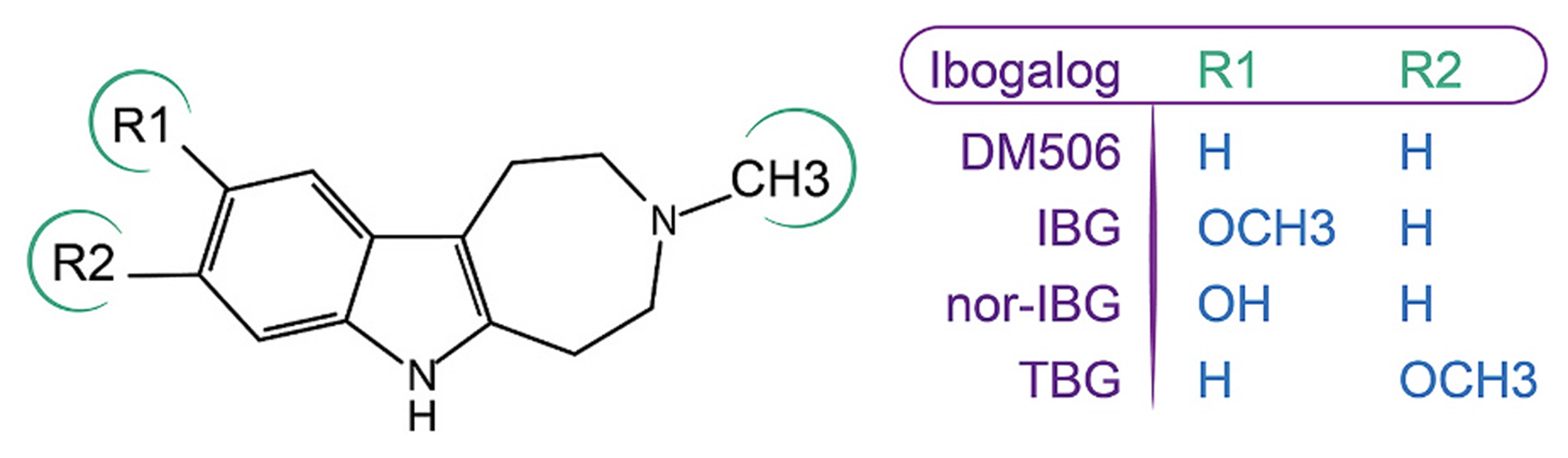
Molecular structures of DM506 (ibogaminalog), IBG (ibogainalog), nor-IBG, and TBG (tabernanthalog).

Novel psychoplastogens derived from iboga alkaloids, so-called “ibogalogs” (see molecular structures in Fig. 1), modulate nearly all members of the serotonin receptor family with different levels of potency and effectiveness [13–18]. For example, tabernanthalog (TBG), ibogainalog (IBG), and ibogaminalog (DM506) activate the 5-HT_2A/2C/6_R subtypes with potencies in the nanomolar concentration range. From a preclinical perspective, the administration of ibogalogs to rodents reduces pain, depression, and anxiety-like behaviors [13–18], which may attenuate the comorbidities associated with seizure. Based on their pharmacological profiles, including potent agonistic activity at the 5-HT_2A_R and reduced ibogalog effects in the presence of selective antagonists, these laboratories identified 5-HT_2A_R activation as the primary mechanism underlying ibogalog-induced behavioral changes in mice. Since these compounds behave as potent agonists of both 5-HT_2A/2C_Rs, it is plausible that the activation of any of these receptor subtypes may decrease the frequency of epileptiform activity in the hippocampus. To investigate this hypothesis, the antiseizure activities of IBG, nor-IBG, and DM506 were evaluated in mice after acute and repeated treatments using the pentylenetetrazol (PTZ)-induced seizure test, as described previously [19,20]. To determine the underlying mechanisms involved in the antiseizure activity of ibogalogs, different approaches were used: (a) examining how various selective antagonists influence the antiseizure activity of ibogalogs; (b) assessing whether the effects of PTZ on the levels of monoamines and related compounds were modified by ibogalogs after acute and repeated treatments; (c) determining whether ibogalogs can suppress epileptiform discharges induced by kainic acid (KA) in rat hippocampal slices [21,22]; and (d) estimating potential receptor candidates, including 5-HT_2A/2B/2C_R and 5-HT_1A/1D_R subtypes, for the observed activity of ibogalogs. In accordance with the International League Against Epilepsy (ILAE) recommendations [23], we used the term antiseizure to refer to the suppression of seizure-like activity, and avoided the term antiepileptic because disease modification (antiepileptogenesis/epileptogenesis) was not evaluated in this study.

## 2. Material and Methods

### 2.1. Material

Revvity (Waltham, MA, USA; Zurich, Switzerland) supplied recombinant HEK293 cells that overexpressed human 5-HT_1A_R, along with the HTRF IP-One Gq Detection Kit and Unifilter^®^ GF/C plates. The HEK293- T cell line was obtained from the American Type Culture Collection (ATCC^®^) in Manassas, VA, USA. NIH/3T3 cells, serotonin hydrochloride (5-HT), pargyline hydrochloride, ascorbic acid, volinanserin, and SB242084 dihydrochloride hydrate were acquired from Merck (Buchs, Switzerland) and TargetMol Chemicals Inc. (Wellesley Hills, MA, USA). WAY100635 maleate was obtained from TOCRIS Bioscience (Minneapolis, MN, USA). The GloSensor was purchased from Promega (Madison, WI, USA). Polyethyleneimine was purchased from Merck (Buchs, Switzerland) and Alfa Aesar (Ward Hill, MA, USA). Clear-bottom 384-well plates with a poly L-lysine coating were sourced from Greiner Bio One (Monroe, NC, USA). D-luciferin was procured from Gold Biotechnology Inc., based in St. Louis, MO, USA. Opti-MEM plates were sourced from ThermoFisher Scientific, located in Waltham, MA, USA. Scintillation vials with a capacity of 6 mL, along with Ultima Gold and MicroScint-20 or –PS scintillation cocktails, were acquired from PerkinElmer in Schwerzenbach, Switzerland.Penicillin, streptomycin, Dulbecco’s modified Eagle medium (DMEM), and fetal bovine serum (FBS) were sourced from Corning Cellgro located in Manassas, VA, USA. Pentylenetetrazol (PTZ) was acquired from Sigma-Aldrich, with locations in St. Louis, MO, USA, and Saint Quentin Fallavier, France. Kainic acid (KA) was procured from Hello Bio Inc. in Avonmouth, Bristol, UK. Ibogainalog hydrochloride (IBG), nor-IBG (base), ibogaminalog (hydrochloride and base) (DM506), and tabernanthalog fumarate (TBG) were synthesized by Ambeed, Inc. in Arlington Hts, IL, USA, according to previously established protocols [13,24]. Salts, bases, buffers, and solvents were purchased from commercial suppliers and used as received.

### 2.2. Animals

All experimental procedures adhered to the guidelines set by the National Institute of Health for the Care and Use of Laboratory Animals. These procedures received approval from the Regional Ethics Committee for Animal Experimentation and were conducted in compliance with the European Communities Council Directive (86/609/EEC + 2010/63/UE) as well as the Institutional Animal Care Committee (Animal Experimentation Ethics Committee, 054, 202, France). The design and implementation of the experiments followed the ARRIVE guidelines 2.0.

Adult male C57BL/6J mice (aged 6 weeks and weighing 30–35 g) and male Wistar rats (aged 4–6 weeks and weighing 100–150 g) were obtained from Janvier Labs (Le Genest Saint Isle, France) and Charles River Laboratories (Gottingen, Germany), respectively. The animals were housed in groups in standard cages with unrestricted access to a regular laboratory diet and tap water. They were kept in a ventilated room maintained at a temperature of 22 ± 1 °C, with a 12-hour light and 12-hour dark cycle, with lights on from 7:00 a.m. to 7:00 p.m. Six weeks-old mice were selected as young adults to balance physiological maturity with experimental consistency and manageable inter-individual variability [25]. Four to six weeks-old rats were used because this age range is commonly chosen to optimize slice viability, preservation of hippocampal circuitry, and stability of network-level recordings [26].

### 2.3. Antiseizure test in mice

**Fig. 2.**
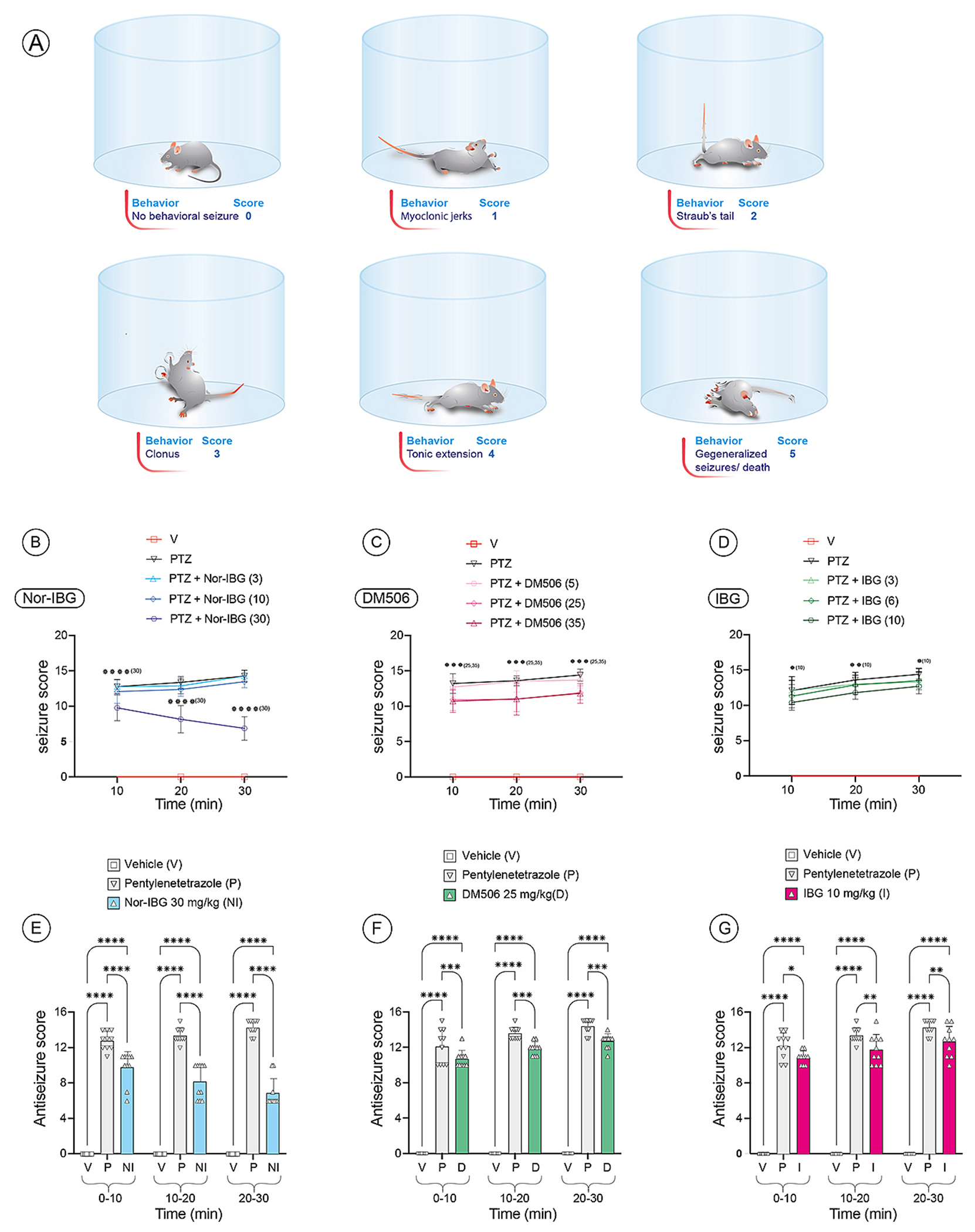
The acute antiseizure activity of ibogalogs was assessed in male mice using the PTZ test. (A) Diagram depicting the range of seizure types encountered during PTZ administration and scoring of each distinct behavior. (B-D) Antiseizure effects of nor-IBG (3, 10, and 30 mg/kg) (B), DM506 (5, 25, and 35 mg/kg) (C), and IBG (3, 6, and 10 mg/kg) (D) in PTZ (P)-treated mice during the entire testing time (0–30 min). (E-G) Bar charts illustrating the acute antiseizure activities of nor-IBG (NI) (30 mg/kg) (E), DM506 (D) (25 mg/kg) (F), and IBG (I) (10 mg/kg) (G) at each 10-min interval after PTZ injection. Analysis using two-way ANOVA and Tukey’s post hoc test on the data (mean ± SEM; n = 10 per condition) indicated that PTZ induced seizure activity during the entire testing period, compared to vehicle (V)-treated animals (****p < 0.0001). Antiseizure scores across the 10-min period were as follows: nor-IBG (****p < 0.0001) and DM506 (***p < 0.001) scores were consistent across all time periods, whereas IBG scores during the 10–30 min period (**p < 0.01) were significantly higher than those in the 0–10 min period (*p < 0.05).

The antiseizure properties of IBG, nor-IBG, and DM506 (base) (refer to molecular structures in Fig. 1) were evaluated in mice using the PTZ-induced seizure test as previously outlined [15,19] (Fig. 2). Male mice (n = 10/condition) were acclimated to the experimental environment for 24 h prior to the commencement of the experiment. The following day, the mice received intraperitoneal injections of varying doses of DM506 (5, 25, and 35 mg/kg), IBG (3, 6, and 10 mg/kg), nor-IBG (3, 10, and 30 mg/kg), each dissolved in 1% DMSO, or a vehicle. Antiseizure effects were evaluated 30 min post-administration. These specific doses were selected based on earlier research that demonstrated beneficial behavioral effects without adverse reactions [14–17]. Mice that had been pretreated were administered an intraperitoneal injection of 70 mg/kg PTZ, freshly prepared in 0.9% NaCl, to trigger seizures. To assess whether a subthreshold dose of DM506 (5 mg/kg) or nor-IBG (3 mg/kg) could produce antiseizure effects following repeated administration, groups of mice (n = 10 for each condition) received injections for 14 consecutive days, and the PTZ test was conducted on days 7 and 14, respectively. To evaluate the involvement of 5-HT_2A_R and 5-HT_2C_R, the selective antagonists volinanserin (0.05 mg/kg, s.c.) and SB242084 (0.5 mg/kg, i.p.), each dissolved in saline, were administered 15 min prior to each ibogalog [27,28] were administered 15 min prior to each ibogalog [14,28].

The animals were subsequently placed in clear Plexiglass cylinders with a diameter of 30 cm, which were open at the top to ensure adequate ventilation of the animals. Their behavior was monitored for 60 min, during which the onset, intensity, and type of seizures were documented using a scoring system adapted from[19] and outlined in Fig. 2: score 0, indicates no observable behavioral seizures; score 1, represents myoclonic jerks, which are brief involuntary body movements; score 2, is characterized by Straub’s tail, where the tail is held rigidly upright; score 3, involves clonus, where the front limbs bend with or without the loss of the standing reflex, and the animals may make bicycle-like motions with the front limbs, possibly accompanied by a loss of the righting reflex; score 4, is defined by tonic extension, where the hind limbs are extended backward and parallel to the ground due to tonic contraction; and score 5, signifies generalized seizures leading to death. The total score was determined by summing the five individual behavioral scores, each ranging from 1 to 5, and dividing the result by the number of animals used. Animals that survived this period were considered protected. Thirty minutes after the PTZ injection, all animals, including those in the control group, were euthanized using general anesthesia with isoflurane (3%, flow rate 0.8 L/min), and decapitated in accordance with regulatory guidelines.

### 2.4. Determination of monoamines and related compounds in the hippocampus

To assess the potential interaction between PTZ and ibogalogs within neurotransmitter systems in the hippocampus, the levels of monoamines and their metabolites were measured in the dorsal hippocampus (DH), as previously described [20]. Mice received an acute intraperitoneal injection of 30 mg/kg nor-IBG, 25 mg/kg DM506, or a vehicle. Another set of mice was injected daily (i.p.) with 3 mg/kg nor-IBG, 5 mg/kg DM506, or vehicle for 14 days. Thirty minutes after the final injection, the mice were administered 70 mg/kg PTZ or a vehicle. The mice were randomly assigned to receive either ibogalog or a vehicle as pretreatment, and either PTZ or a vehicle as treatment. Thirty minutes later, the rats they were euthanized by cervical dislocation. Their brains were swiftly extracted, immersed in cold isopentane for approximately two minutes at −35 °C, and then stored at −80 °C until dissection. Brain dissection was conducted in the frontal plane using a cryostat set at −24 °C. The DH was extracted using an 800 μm diameter stainless-steel punch, and a bilateral tissue sample was placed into a pre-weighed, labeled 0.6 mL tube [29]. The tube was stored at −80 °C until neurochemical analysis.

The levels of DA and its metabolite 3,4-dihydroxyphenylacetic acid (DOPAC), 5-HT and its metabolite 5-hydroxyindole-3-acetic acid (5-HIAA), and NE were measured using high-pressure liquid chromatography (HPLC) with electrochemical detection, as previously described [20]. Brain tissue samples from a specific region were removed from a deep freezer, placed on ice, swiftly cleaned, and weighed using a precision balance. The tissues were then homogenized in 100 μL of 0.1 N HClO4 by sonication. The samples were centrifuged at 13,000 rpm for 30 min at 4 °C. A 20 μL portion of the supernatant was injected into the HPLC system using a manual injector (Rheodyne 7725i, C.I.L.-Cluzeau, Sainte-Foy-La-Grande, France) on an Equisil ODS (C18) HPLC column (150 x 4.6 mm, 5 μm; C.I.L.-Cluzeau). The mobile phase was supplied at a flow rate of 1.2 mL/min using an HPLC pump LC20-AD from Shimadzu, France. The buffer consisted of 60 mM NaH2PO4, 0.1 mM disodium EDTA, and 2 mM octane sulfonic acid, along with 7% methanol. The pH was adjusted to 4.0 using orthophosphoric acid, and the solution was filtered through a 0.22 mm filter (Millipore) filter. The column temperature was maintained at 40 °C. A colorimetric cell (Cell 5011, ESA, Paris, France) in conjunction with a programmable detector (Coulochem II, ESA) was used to identify the monoamines and related compounds. [30]. The electrodes were adjusted to potentials of + 350 mV and −270 mV, respectively. The output signals were collected using an interface (ULYSS, Toulouse, France) linked to a computer running AZURE software (Toulouse, France). Calibration curves were established once the peaks for the various compounds of interest were distinctly separated, with final adjustments made using sodium octyl sulfonate and phosphoric acid. Adjustments in the detector gain throughout the chromatogram enabled the selection of suitable gain settings for each compound and brain region [31]. The sensitivities of NE, DOPAC, DA, 5-HIAA, and 5-HT were approximately 5, 3, 3, 6, and 10 pg/20μ L, respectively, with a signal/noise ratio of 3:1. Each day, standard solutions at a concentration of 1 ng/10 μL were injected both before and after a sequence of samples. In the experimental group, HPLC injections were performed without prior knowledge of the sample details. Neurochemical results are reported as pg/mg of tissue.

### 2.5. Electrophysiological recording in hippocampal slices

To evaluate the antiseizure properties of ibogalogs, the effects of nor-IBG and DM506 were examined in hippocampal slices exposed to KA, as described previously [20]. These slices included the CA3c area of the hippocampus, which serves as a significant intrahippocampal origin of local field potentials and exhibits high-amplitude oscillatory activity [32]. Initially, each animal was anesthetized using isoflurane before decapitation. The brain was swiftly extracted and immersed in cold (3–5 °C) and oxygenated (95% O_2_ + 5% CO_2_) artificial cerebrospinal fluid (aCSF), which contained 121 mM NaCl, 5 mM KCl, 2.5 mM CaCl2, 1.25 mM KH2PO4, 1.3 mM MgSO4, 26 mM NaHCO3, and 10 mM glucose, with a pH of 7.4. Before each experiment, aCSF was freshly prepared using pre-filtered deionized water. Transverse acute hippocampal slices (approximately 500 μm thick), were prepared from both hippocampi using a Stoelting tissue slicer (Wood Dale, IL, USA). After dissection, the hippocampal slices were initially incubated in oxygenated aCSF at approximately 20 °C for 45 min. Following this, the slices were transferred to a gas–liquid interface recording chamber, supported by a nylon mesh, where they were continuously perfused with oxygenated and preheated (35 °C) aCSF at a flow rate of 1 mL/min for 50 min. In all experimental groups, persistent and consistent epileptiform discharges were triggered in hippocampal slices by administering 0.5 μM KA (dissolved in aCSF). Following the recording of stable KA-induced epileptiform activity for 10 min, a bath perfusion of nor-IBG or DM506 (10 and 50 μM; dissolved in aCSF) was simultaneously applied with 0.5 μM KA for additional 30–40 min. To determine the role of 5-HT_2A_Rs, 1 μM volinanserin (dissolved in aCSF) was perfused alone or in the presence of 50 μM nor-IBG. The selected concentrations demonstrated optimal performance compared with the other combinations evaluated.

Extracellular local field potential (LFP) recordings were conducted using glass electrodes (3–5 MΩ, Kwik-Fil capillaries WP Instruments, Sarasota, FL, USA) relative to the ground. The electrodes were positioned in the CA3c area of the hippocampus using a motorized micromanipulator (IVM-1000; Scientifica, Uckfield, UK). The recorded signals underwent band-pass filtering (0.001–0.3 kHz) and were amplified by a factor of 1000 using a P-511 preamplifier from Grass-Astromed, located in West Warwick, RI, USA. These processed signals were then saved onto a computer hard drive via a CED-1401 data acquisition interface (Cambridge Electronic Design, Cambridge, UK). Computer analysis was conducted on 2-min segments of LFPs captured at intervals of 10, 20, 30, and 40 min following the start of perfusion. These segments underwent offline spectral analysis using the Spike 2.7 software (Cambridge Electronic Design, Cambridge, UK). The frequency of epileptiform discharges was meticulously examined across three 20-s recording segments (technical repetitions) beginning at 40, 70, and 100 s of each recording, which were chosen from each 2-min segment.

### 2.6. Functional activity of ibogalogs at 5-HT_1A/1D_R and 5-HT_2A/2B/2C_R subtypes

Considering that the pharmacological effects of TBG and IBG, but not nor-IBG, have already been established have already established the pharmacological effects of TBG and IBG, but not nor-IBG, at 5-HT_2/6_R subtypes, but not 5-HT_1_Rs [14,16,17], the activity of nor-IBG was assessed at 5-HT_2A/2B/2C_Rs, and the activities of nor-IBG, IBG, TBG, and DM506 at the 5-HT_1A/1D_Rs. Measurement of inositol monophosphate 1 (IP1) accumulation following the activation of 5-HT_2_Rs was conducted using the Homogeneous Time-Resolved Fluorescence (HTRF) IP-One Gq Detection kit, as previously outlined [14–16]. In summary, NIH/3T3 cells were either stably transduced with 5-HT_2A_R or 5-HT_2C_R, or transfected with 5-HT_2B_R, and then plated at a concentration of 3,000 cells/well in Opti-MEM medium. Subsequently, nor-IBG was introduced at concentrations ranging from 0.1 to 1,000 μM, and the plates were incubated for 90 min at 37 °C. This was followed by a 60-min incubation at room temperature with Anti-IP1-Cryptate and IP1-d2, using the IP-One Gq kit. To assess whether the function of the 5-HT_2A/2B/2C_Rs was modified after prolonged exposure to an ibogalog, NIH/3T3 cells were pre-incubated with 0.1 and 1 μM nor-IBG for 120 h, and the activity of 5-HT was subsequently determined.

The activation of heterotrimeric Gi1 (Gαi1/Gβ3/Gγ9) through 5-HT_1A/1D_R was assessed using TRUPATH reporter constructs as previously described [33]. Assays were conducted as previously described [34,35]. In summary, HEK293-T cells were transfected with receptor, RLuc-Gα, Gβ, and eGFP-Gγ constructs using polyethyleneimine at a 1:1:1:1 ratio (human 5-HT_1A_ or 5-HT_1D_:Gα:Gβ:Gγ). Typically, 6–7 million cells in a 10 cm dish containing DMEM with 1% (v/v) dFBS were transfected with a total of 8 μg of DNA. The next day, 20,000 cells per well were seeded into 384-well plates with white bottoms that had been coated with poly L-lysine, using DMEM with 1% dFBS. The following day, the medium was removed, and the cells were rinsed with HBSS containing 20 mM HEPES (pH 7.4). Subsequently, 15 μL of drug solutions at a 3x concentration were mixed with 30 μL of HBSS containing 20 mM HEPES (pH 7.4), 0.1% (w/v) FBS, and 0.01% (w/v) ascorbic acid. The cells were then incubated at 37 °C for 30 min, after which 15 μL of freshly prepared 30 μM coelenterazine 400a was added. The plates were promptly analyzed using a Victor NIVO plate reader (Perkin Elmer), employing emission filters of 395 nm for RLuc8-coelenterazine 400a and 510 nm for GFP2, with an integration time of 1 s per well. The Bioluminescence Resonance Energy Transfer (BRET) ratios were calculated by dividing the GFP2 emission by the RLuc8 emission. To assess the antagonistic effects of ibogalogs, the activity of 100 nM 5-HT was tested against increasing concentrations of each ibogalog, and the resulting inhibitory effects were compared with those of the strong 5-HT_1A_R antagonist, WAY100635 [36].

### 2.7. Statistical analysis

The experimental results were processed using Prism software (GraphPad 10.4.0; Software Inc., La Jolla, CA, USA). All data are expressed as the mean ± SEM. Because treatment effects varied across time bins and endpoints, comparative statements were restricted to within-assay analyses. Time-course experiments were analyzed using two-way ANOVA with treatment as the between-subject factor and time bin (0–10, 10–20, and 20–30 min) as the within-subject repeated-measures factor. When appropriate, post-hoc multiple comparisons were performed within each time bin using Tukey’s correction. The Full ANOVA results, including the main effects of treatment and time and treatment × time interaction, are provided in Table 1. Adjusted p values were included in each graph legend. Outliers were objectively removed using the Grubbs test. To compare the slopes of each ibogalog, an unpaired *t*-test was conducted. A p-value of less than 0.05 was considered statistically significant.

## 3. Results

### 3.1. Ibogalogs induce acute antiseizure activity in PTZ-treated mice

Different seizure behaviors appeared a few seconds after PTZ injection (Fig. 2A). Myoclonic jerks were the first manifestations followed by Straub’s tail and clonus. These three behaviors alternated with the dominant myoclonic jerks. As seizure-like manifestations were progressively observed after 10 min, seizure behaviors were analyzed every 10-min period for 30 min.

Two-way ANOVA and Tukey’s post-hoc analysis of the results (mean ± SEM; n = 10 per condition) showed that PTZ significantly increased seizure scores in male mice at each 10-min period tested, compared to vehicle-treated animals (p < 0.0001) (Fig. 2B–G). Subsequently, the acute antiseizure activity of nor-IBG (3, 10, and 30 mg/kg) was assessed (Fig. 2B), and DM506 (5, 25, and 35 mg/kg) (Fig. 2C), and IBG (3, 6, and 10 mg/kg) (Fig. 2D) was assessed in PTZ-treated mice at each 10-min period. Statistical analysis demonstrated significant effects of treatment and time [F_(4, 135)_ = 759.1; p < 0.0001] for all three compounds. Nevertheless, DM506 at a higher dose (35 mg/kg) did not improve the antiseizure effect elicited at 25 mg/kg (p > 0.05). Further Tukey’s post-hoc analysis revealed that 30 mg/kg nor-IBG [F_(2,81)_ = 1022; p < 0.0001] (Fig. 2E) and 25 mg/kg DM506 [F_(2, 81)_ = 1998; p = 0.0002] (Fig. 2F) significantly reduced PTZ-induced seizure scores at each 10-min interval compared to the PTZ-treated group. In contrast, IBG (10 mg/kg) significantly decreased PTZ’s effects in a timeframe-dependent manner: the antiseizure effect at the 10–20 and 20–30 min periods [F_(2, 81)_ = 1454; p = 0.0028; for both] was higher than that at the 0–10 min period [F_(2, 81)_ = 1454; p = 0.0185] (Fig. 2G). However, the reduced scores did not reach the values observed in vehicle-treated mice [F_(2, 81)_ = 1454; p < 0.001]. These findings suggest that ibogalogs trigger immediate antiseizure effects that vary with dosage and timing.

### 3.2. Effect of selective antagonists in the acute antiseizure activity of ibogalogs

**Fig. 3.**
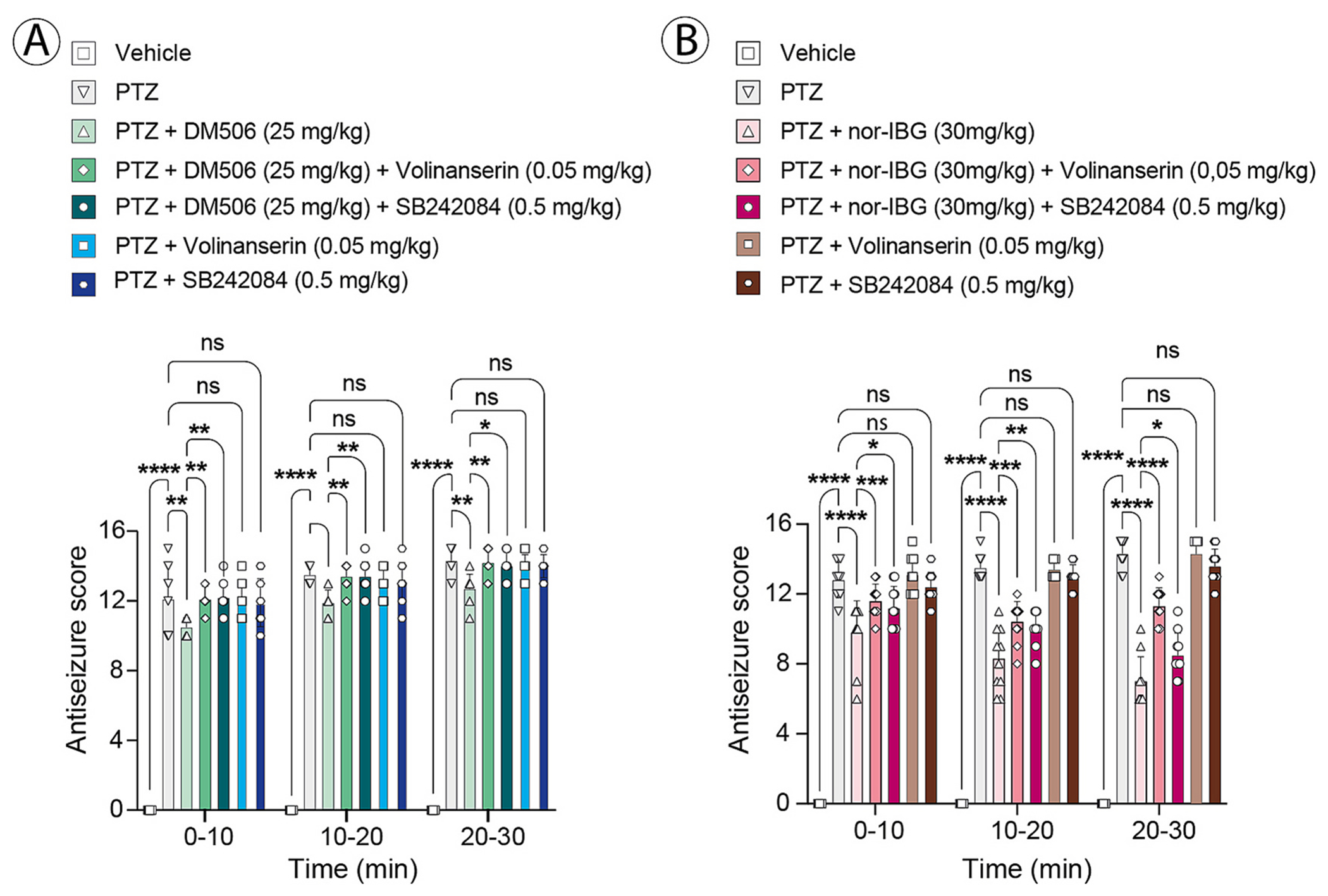
Effects of selective antagonists on the acute antiseizure activity of ibogalogs. Mice (n = 10 per condition) received volinanserin (0.05 mg/kg, s.c.; 5-HT_2A_R antagonist) or SB242084 (0.5 mg/kg, i.p.; 5-HT_2C_R antagonist) 15 min before administration of DM506 (25 mg/kg, i.p.; A) or nor-IBG (30 mg/kg, i.p.; B) in the PTZ model. Bar graphs show seizure scores (mean ± SEM) for each 10-min time bin (0–10, 10–20, 20–30 min). Data were analyzed using two-way ANOVA (treatment × time); full ANOVA results (main effects and interaction) are reported in Table 1. Post-hoc multiple comparisons were performed using Tukey’s test within each time bin. (A) Representative post-hoc comparisons showed that DM506 significantly reduced PTZ-induced seizure scores at 0–10 min (PTZ *vs* PTZ + DM506: p = 0.0013), whereas this effect was abolished by volinanserin (PTZ + DM506 *vs* PTZ + DM506 + volinanserin: p = 0.9999) and significantly attenuated by SB242084 (PTZ + DM506 *vs* PTZ + DM506 + SB242084: p = 0.0005). (B) For nor-IBG, robust antiseizure effects were observed across time bins (PTZ *vs* PTZ + nor-IBG: p < 0.0001), which were significantly reduced by both volinanserin and SB242084 at later time points (e.g., 10–30 min; p < 0.0001). These results indicated combined contributions of both 5-HT_2A_R and 5-HT_2C_Rs.

To determine whether 5-HT_2A_R and/or 5-HT_2C_R contribute to the acute antiseizure activity of ibogalogs, we examined the effects of the selective 5-HT_2A_R antagonist volinanserin (0.05 mg/kg, s.c.) and the selective 5-HT_2C_R antagonist SB242084 (0.5 mg/kg, i.p.) in mice administred PTZ with either DM506 (Fig. 3A) or nor-IBG (Fig. 3B). Data were analyzed using two-way ANOVA (treatment × time). The full ANOVA outputs, including main effects and interaction terms with degrees of freedom, are provided in Table 1, while the main text reports only key effects supporting biological interpretation.

DM506 (25 mg/kg; Fig. 3A): Two-way ANOVA revealed significant main effects of treatment [F_(6,189)_ = 937.4; p < 0.0001] and time [F_(2,189)_ = 73.45; p < 0.0001, as well as a significant treatment × time interaction [F_(12,189)_ = 2.169; p = 0.0147] (Table 1). Post-hoc Tukey comparisons performed within each time bin showed that DM506 significantly reduced PTZ-induced seizure scores across the time course, and that co-administration of either volinanserin or SB242084 attenuated this antiseizure effect, consistent with receptor-mediated blockade (Prism-adjusted p values; see figure legend).

Nor-IBG (30 mg/kg; Fig. 3B): Two-way ANOVA showed a significant main effect of treatment [F_(6189)_ = 649.9; p < 0.0001] and a significant treatment × time interaction [F_(12,189)_ = 8.272; p < 0.0001], whereas the main effect of time was not significant [F_(2,189)_ = 2.027; p = 0.1345] (Table 1). Nevertheless, neither ibogalog restored seizure scores to vehicle levels over the 10–40 min interval, indicating partial protection under these experimental conditions. Tukey post-hoc comparisons within each time bin confirmed a robust antiseizure effect of nor-IBG against PTZ, and demonstrated that both volinanserin and SB242084 reduced nor-IBG effect in a time-dependent manner, supporting the involvement of 5-HT_2A_R and 5-HT_2C_R signaling in the acute response (Prism-adjusted p values; see figure legend). Collectively, these acute PTZ data are consistent with involvement of both 5-HT_2A_R and 5-HT_2C_Rs in ibogalog-mediated seizure suppression.

### 3.3. Repeated administration of a subthreshold dose of ibogalog enhances its antiseizure effectiveness

**Fig. 4.**
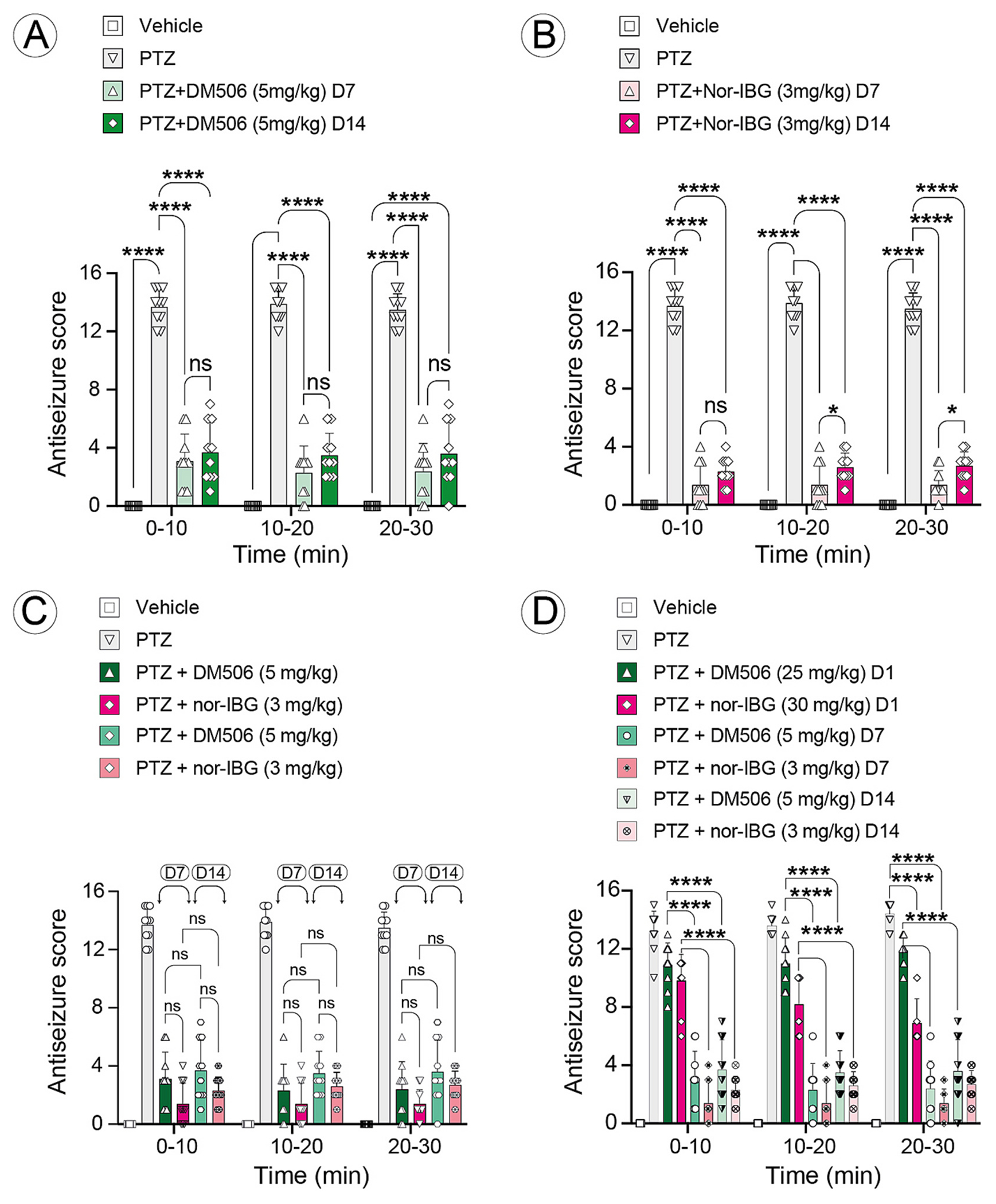
Antiseizure effects following repeated administration of a subthreshold dose of DM506 or nor-IBG in PTZ-treated mice. Groups of mice (n = 10 per condition) received daily intraperitoneal injections for 14 days of a subthreshold dose of DM506 (3 mg/kg) or nor-IBG (5 mg/kg). Antiseizure scores (mean ± SEM) were assessed on day 7 (D7) and day 14 (D14) and are shown for each 10-min time bin (0–10, 10–20, 20–30 min). Data were analyzed using two-way ANOVA (treatment × time); full ANOVA results are provided in Table 1. Post-hoc multiple comparisons were performed using Tukey’s test within each time bin. (A,B) Repeated administration of DM506 (p < 0.0001 at 0–10, 10–20, and 20–30 min) (A) or nor-IBG (p < 0.0001 at at 0–10, 10–20, and 20–30 min) (B) significantly reduced PTZ-induced seizure activity at both D7 and D14 (PTZ *vs* PTZ + ibogalog). (C) No significant difference between D7 and D14 was observed for DM506 (p = 0,9207) or nor-IBG (0,6746), indicating a stable antiseizure effect over time. (D) Direct comparison of acute *vs* repeated administration demonstrated a significantly greater antiseizure effect after repeated treatment (p < 0.0001).

To test whether repeated dosing with a subthreshold amount of each ibogalog increases or not its antiseizure efficacy, mice received DM506 (5 mg/kg/day) (Fig. 4A) or nor-IBG (3 mg/kg/day) (Fig. 4B) for 14 days, and the antiseizure activity was assessed on days 7 and 14. Two-way ANOVA (treatment × time), and full ANOVA outputs are provided in Table 1.

DM506 administred repeatedly (Fig. 4A): Two-way ANOVA showed a strong main effect of treatment [F_(3,108)_ = 513.7; p < 0.0001], with neither a significant time effect [F_(2,108)_ = 0.3317; p = 0.7184] nor a treatment × time interaction [F_(6,108)_ = 0.2685; p = 0.9505] (Table 1), indicating a stable treatment effect across time bins. Tukey comparisons within each time bin confirmed that repeated DM506 administration reduced PTZ-induced seizure scores relative to PTZ alone on days 7 and 14 (Prism-adjusted p-values; see figure legend).

Nor-IBG administred repeatedly (Fig. 4B): Statistical analysis indicated a significant main effect of treatment [F_(6,189)_ = 649.9; p < 0.0001] and a significant treatment × time interaction [F_(12,189)_ = 8.272; p < 0.0001], while the main effect of time was not significant [F_(2,189)_ = 2.027; p = 0.1345] (Table 1). Tukey comparisons within each time bin indicated that repeated nor-IBG dosing significantly reduced PTZ-induced seizure scores compared to PTZ alone across the time course (Prism-adjusted p values; see figure legend). Taken together, repeated administration of subthreshold doses produced strong antiseizure effects for both ibogalogs. Because effects can vary by endpoint and sampling window, we report comparisons within each panel ibogalog and time bin and provide complete ANOVA outputs in Table 1.

### 3.4. Effect of ibogalogs on hippocampal tissue content of monoamines

**Fig. 5.**
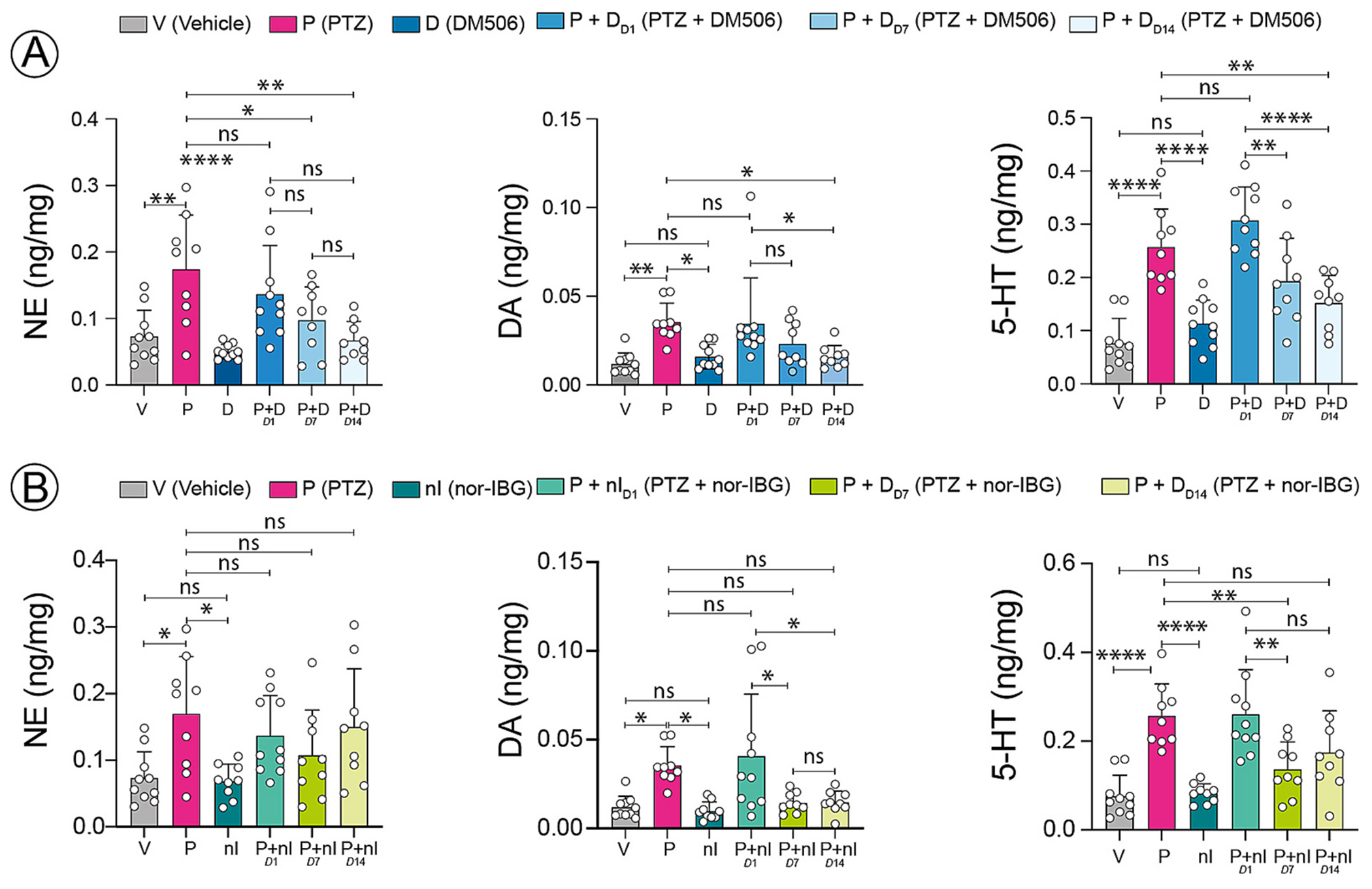
Effect of ibogalogs on hippocampal tissue content of monoamines. (A,B) PTZ (P) significantly increased the tissue content of 5-HT (****p < 0.0001) more than that of NE and DA (p* < 0.05; **p < 0.01), compared to the vehicle (V) group. Acute treatment (D1) with any ibogalog did not alter basal monoamine levels or modify PTZ effects. (A) Subchronic DM506 (D) treatment reduced the PTZ-induced increase in NE (*p < 0.05) after 7 days (D7) and in NE (**p = 0.01), DA (*p = 0.05), and 5-HT (**p = 0.01) after 14 days (D14). (B) Subchronic nor-IBG (nI) treatment attenuated the PTZ-induced increase in DA after 7 and 14 days (*p = 0.05) and 5-HT after 7 days (**p = 0.01), with no significant (ns) effect on NE.

To explore the potential link with the antiseizure properties of ibogalogs, we examined the impact of DM506 (Fig. 5A) and nor-IBG (Fig. 5B) on monoamine levels in hippocampal tissue obtained from PTZ-treated mice. PTZ significantly enhanced 5-HT tissue content [F_(5, 51)_ =20.46; p <0.0001] more than NE [F_(5, 51)_ = 7.501; p < 0.0016] and DA tissue content [F_(5, 51)_ = 5.409; p = 0.0049], compared to the vehicle group, similar to a previous study [20]. Acute treatment with DM506 modified neither the basal levels of monoamines nor the effect of PTZ (Fig. 5A). Nonetheless, subchronic treatment with a subthreshold dose of DM506 significantly reduced the acute effect of PTZ on NE levels [F_(5, 51)_ = 7.501; p = 0.0388] after 7 days, and on NE [F_(5, 51)_ = 7.501; p = 0.0011], DA [F_(5, 51)_ = 5.409; p = 0.0369], and 5-HT levels [F_(5, 51)_ = 20.46; p = 0.0072] after 14 days. These effects were similarly observed on DOPAC and 5-HIAA levels, the respective metabolites of DA and 5-HT (data not shown).

Similar to DM506, acute injection of nor-IBG was devoid of activity on its own on hippocampal monoamine levels and did not counteract the effect of PTZ [F_(5, 50)_ = 3.886; p = 0.8785]. However, after subchronic treatment, a subthreshold dose of nor-IBG significantly reduced the ability of PTZ to enhance DA [F_(5, 50)_ = 6.486, p = 0.0104 (day 7); p = 0.0118 (day 14)] or 5-HT tissue content [F_(5, 50)_ = 12.39; p = 0.0055 (day 7)]. The metabolite DOPAC followed the same response as DA, but 5-HIAA content was similar in all groups receiving PTZ with DM506 or nor-IBG. No change in the effect of PTZ on NE content was observed upon subchronic injection of nor-IBG. In conclusion, subchronic, but not acute, treatment reduced PTZ-evoked monoamine alterations, with compound- and analyte-specific patterns.

### 3.5. DM506 and nor-IBG suppress kainate-induced epileptiform activity in hippocampal slices

**Fig. 6.**
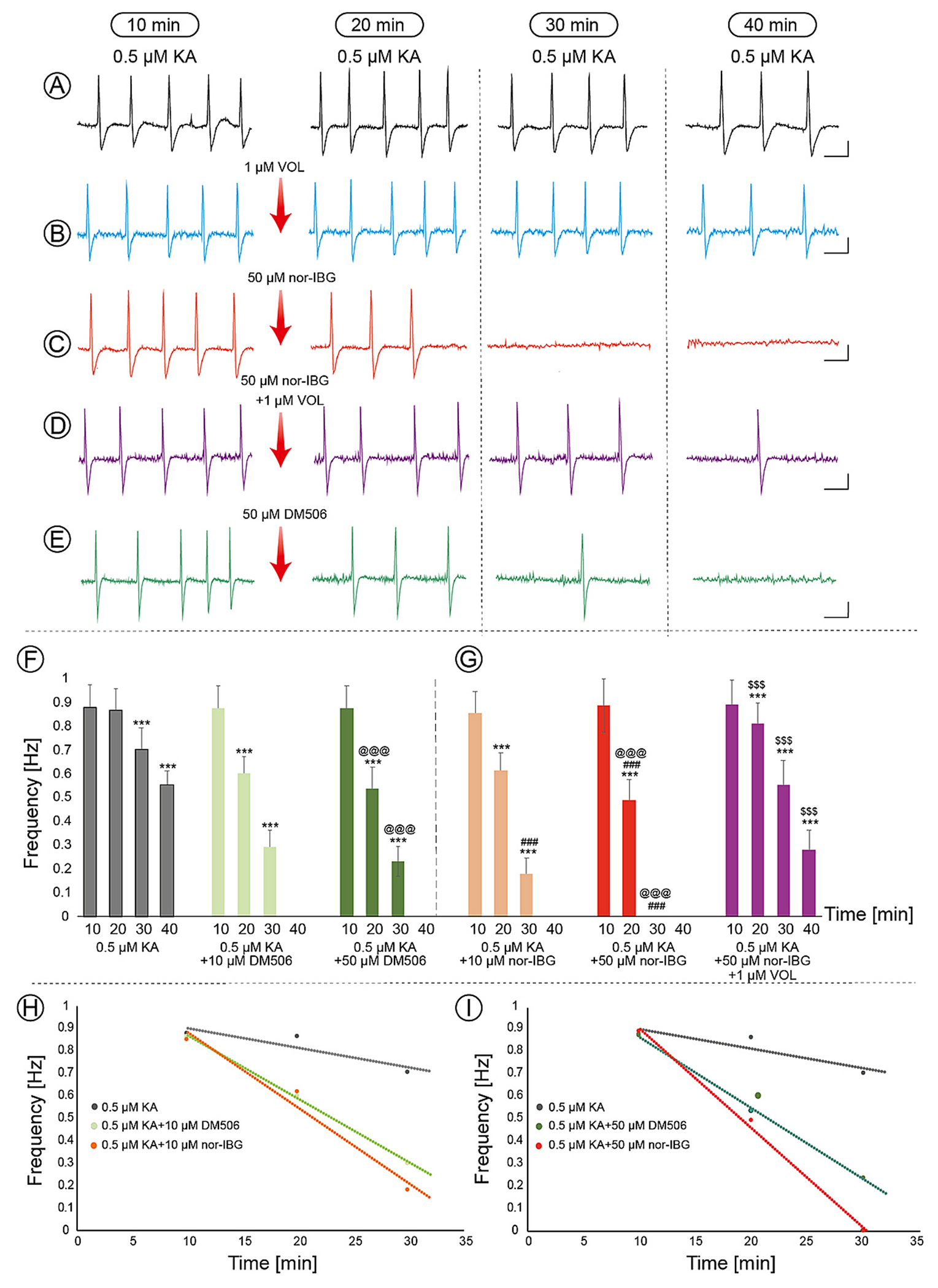
Impact of ibogalogs on KA-triggered epileptiform activity in the hippocampal CA3c region. (A,B) Epileptiform activity caused by 0.5 μM KA alone (A) was comparable to that observed with 0.5 μM KA combined with 1 μM volinanserin (B). (C,E) Suppressive effect of 50 μM nor-IBG (C) and 50 μM DM506 (E) on KA-induced epileptiform activity. Arrows indicate ibogalog perfusion after 10 min of administration. (D) Volinanserin attenuates the suppressive effect of nor-IBG on KA-induced epileptiform discharges. Calibration: 500 μV and 1 s. (F,G) One-way ANOVA and Tukey’s post-hoc test analysis of the data (mean ± SD) revealed that KA triggered epileptiform activity in a time-dependent manner, with a notable reduction in the mean frequency observed at 30 and 40 min compared to 10 min (***p < 0.001). Ibogalogs significantly reduced the mean frequency induced by KA in a concentration-dependent manner, with 50 μM inducing a more pronounced effect than 10 μM (^@@@^p < 0.001 for both). More specifically, 50 μM DM506 (F) significantly reduced this activity at 20–30 min (^###^p < 0.001) and completely blocked the discharges at 40 min, whereas 50 μM nor-IBG (G) significantly reduced the mean frequency induced by KA at 20 min (***p < 0.001) and completely blocked the discharges at 30–40 min. The effect of 50 µM nor-IBG at 20 min was significantly more pronounced than that of 50 μM DM506 (^###^p < 0.001). Both ibogalogs (10 μM) also significantly decreased KA-induced epileptiform activity at 20–30 min (***p < 0.001 for both). Volinanserin significantly counteracted the effects of 50 µM nor-IBG (^$^p<0.001). (H, I) Linear regression for KA-induced epileptiform discharges (R^2^ = 0.81) (●) after perfusion with 10 μM (R^2^ = 0.97) (●) or 50 μM nor-IBG (R^2^ = 0.99) (●), and with 10 μM (R^2^ = 0.99) (●) or 50 μM DM506 (R^2^ = 0.99) (●), respectively. The slopes (Δfrequency/Δ time) indicate that the frequency decay rates for each ibogalog were faster than those for KA (●). The unpaired *t*-test showed that the slopes of 50 μM nor-IBG and 50 μM DM506 were significantly different (p < 0.0001).

Electrophysiological results obtained from hippocampal slices showed that 0.5 μM KA reliably induced stable epileptiform discharges (n = 7 rats, 26 slices) (Fig. 6A), which served as the control condition for subsequent comparisons. The frequency discharges persisted for up to 40 min in a time-dependent manner: 0.88 ± 0.09 Hz (10 min), 0.86 ± 0.09 Hz (20 min), 0.70 ± 0.09 Hz (30 min), and 0.56 ± 0.06 Hz (40 min) (Fig. 6A,F). One-way ANOVA followed by Tukey’s post-hoc test showed a significant reduction in the average frequency at 30 and 40 min when compared to 10 min (p < 0.001) (Fig. 6F). A similar, but more pronounced, time-dependent decrease in frequency was observed for nor-IBG at 10 and 50 μM (n = 8 rats, 28–29 slices) in the presence of KA (Fig. 6C,G). According to the statistical analysis, the frequency values observed between 20 and 40 min were notably lower than to those recorded at 10 min (Fig. 6G). Nor-IBG (50 μM) significantly decreased the mean frequency induced by KA at 20 min (0.49 ± 0.08 Hz) compared to KA (alone) at 10 min (0.89 ± 0.11 Hz) (p < 0.001) and completely blocked the discharges at 30 and 40 min (Fig. 6C,G). At a lower concentration (10 μM), nor-IBG significantly reduced the mean frequency at 20 min (0.61 ± 0.08 Hz) and 30 min (0.18 ± 0.07 Hz) compared to KA (alone) at 10 min (0.86 ± 0.09 Hz) (p < 0.001 for both), but completely blocked the discharges only at 40 min (Fig. 6G). A similar time-dependent attenuation in frequency was also noted for DM506 (10 and 50 μM; n = 8–9 rats, 27–32 slices) (Fig. 6E,F). DM506 (50 μM) significantly decreased the mean frequency induced by KA at 20 min (0.54 ± 0.09 Hz) and 30 min of recording (0.23 ± 0.06 Hz) compared to KA (alone) at 10 min (0.88 ± 0.09 Hz) (p < 0.001 for both) and completely blocked the discharges at 40 min (Fig. 6E,F). At a lower concentration (10 μM), DM506 significantly reduced the mean frequency at 20 min (0.60 ± 0.07 Hz) and 30 min (0.29 ± 0.07 Hz) compared to KA (alone) at 10 min (0.88 ± 0.09 Hz) (p < 0.001 for both) and completely blocked the discharges at 40 min (Fig. 6F). Using one-way ANOVA followed by Tukey’s post-hoc test, significant differences were identified between ibogalogs at identical concentrations across specific time intervals. The results showed that 10 μM and 50 μM nor-IBG induced a significantly greater reduction in KA-evoked discharges than the corresponding concentrations of DM506 (Fig. 6C,G). Maximal reductions were attained at 30 min (10 μM) and 20 min (50 μM) (p < 0.001 for both). Statistical analysis also showed that 50 μM DM506 and 50 μM nor-IBG significantly decreased the frequency of discharges compared to their respective 10 μM concentrations at 20 and 30 min of recording (p < 0.001 for both times) (Fig. 6F,G).

To assess the role of 5-HT_2A_R in the antiseizure activity of ibogalogs, the selective antagonist volinanserin was used. When volinanserin (1 μM) was combined with KA (n = 7 rats, 24 slices), epileptiform discharges with frequencies of 0.84 ± 0.10 Hz (20 min), 0.68 ± 0.07 Hz (30 min), and 0.52 ± 0.05 Hz (40 min) were obtained (Fig. 6B), which did not differ from those obtained with KA alone (Fig. 6A). In addition, volinanserin (alone) (n = 5 rats, 18 slices) did not elicit any electrophysiological response in the absence of KA either (data not shown). When volinanserin was combined with KA + 50 μM nor-IBG (n = 7 rats, 25 slices), a significant reduction in discharge frequency was observed at 20 min (0.81 ± 0.08 Hz), 30 min (0.56 ± 0.13 Hz), and 40 min (0.28 ± 0.08 Hz) compared to KA (alone) at 10 min (0.89 ± 0.10 Hz) (p < 0.001 for all times) (Fig. 6D). One-way ANOVA and Tukey’s post-hoc analysis of the results showed that volinanserin significantly increased the discharge frequency attenuated by 50 μM nor-IBG during the entire recording (20–40 min) (p < 0.001) (Fig. 6G). These results indicate that nor-IBG–induced suppression of KA-induced epileptiform discharges is mediated by 5-HT_2A_R activation.

To evaluate the effects of concentration and rate on each ibogalog, linear regressions were conducted for both nor-IBG and DM506 (Fig. 6H, I).The linear regression slopes, with a goodness of fit (R^2^) greater than 0.89, indicated that both 10 μM (0.0336 ± 0.0011 Hz/min) and 50 μM nor-IBG (0.0445 ± 0.0009 Hz/min), as well as 10 μM (0.0282 ± 0.0008 Hz/min) and 50 μM DM506 (0.0319 ± 0.0009 Hz/min), reduced the KA-induced discharge frequency at a significantly quicker pace compared to KA alone (0.0086 ± 0.0010 Hz/min). An unpaired *t*-test comparing the slopes of 50 μM nor-IBG and 50 μM DM506 revealed a significant difference in activity between the two ibogalogs (t = 10.11, df = 77.11, p < 0.0001).

### 3.6. Functional activity of ibogalogs at 5-HT_1A/1D_R and 5-HT_2A/2B/2C_R subtypes

**Fig. 7.**
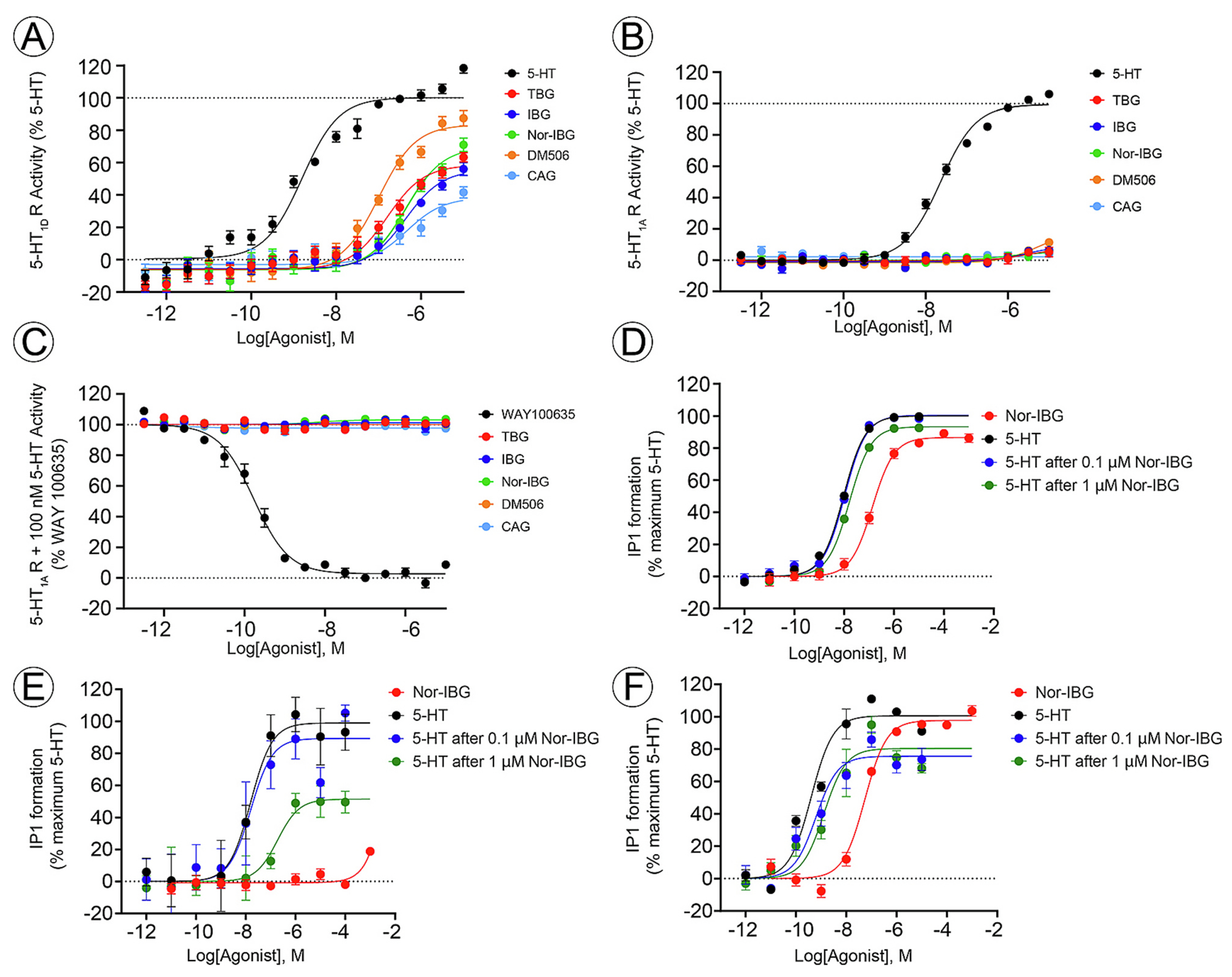
Functional activity of ibogalogs at human 5-HT_1A/1D_R and 5-HT_2A/2B/2C_R subtypes. (A,B) HEK293-T cells transfected with 5-HT_1D_R (A) or 5-HT_1A_R (B) were incubated (30 min) with DM506 (●), IBG (●), nor-IBG (●), TBG (●), and CAG (●), and the activity (mean ± SEM) was compared to that of 5-HT (●). (A) Ibogalogs activated 5-HT_1D_R in the nM concentration range (n = 6). DM506 acted as a full agonist, whereas the other compounds acted as partial agonists. (B) Ibogalogs (10 μM) did not activate 5-HT_1A_R compared to the agonist 5-HT (●) (n = 4). (C) Thus, the antagonistic activity of ibogalogs was assessed against 100 nM 5-HT. The results indicated that ibogalogs do not inhibit this receptor compared to the selective antagonist WAY100635 (●) (n = 4). (D-F) NIH/3T3 cells expressing the 5-HT_2A_R (D), 5-HT_2B_R (E), or 5-HT_2C_R (F) were incubated (90 min) with different concentrations of nor-IBG (●), or pre-incubated (120 h) with 0.1 (●) or 1 μM nor-IBG (●), and the activity of 5-HT (●) was subsequently determined. One-way ANOVA and Bonferroni’s post hoc analysis of the results (mean ± SEM; n = 3–4) indicated that the potency and efficacy of 5-HT at the 5-HT_2A_R (p < 0.005; p < 0.0001) and 5-HT_2B_R (p < 0.05; p < 0.005) were significantly decreased only by 1 μM nor-IBG concentration. In the 5-HT_2C_R, only the 5-HT efficacy was significantly decreased by 0.1 μM or 1 μM nor-IBG (p < 0.005). The calculated EC_50_ and E_max_ values are listed in Table 2.

To complement previous studies on ibogalogs at diverse 5-HT receptor subtypes [14–17], we determined the functional activities of DM506, IBG, nor-IBG, TBG, and CAG at 5-HT_1D_R (Fig. 7A) and 5-HT_1A_R (Fig. 7B,C), and the activity of nor-IBG at the 5-HT_2A_R (Fig. 7D), 5- HT_2B_R (Fig. 7E), and 5-HT_2C_R (Fig. 7F), respectively. Concentration-response curves (mean ± SEM) for 5-HT_1D_R (Fig. 7A) provided the following potency [EC_50_ (half-maximal stimulatory concentration)] sequence: DM506 (110 ± 20 nM) > TBG (209 ± 58 nM) > CAG (407 ± 164 nM) ~ nor-IBG (447 ± 134 nM) > IBG (661 ± 182 nM) (Table 2). The observed efficacies (E_max_) at 10 μM indicated that DM506 (89 ± 3%), nor-IBG (75 ± 7%), TBG (61 ± 7%), IBG (59 ± 12%), and CAG (40 ± 6%) behaved as partial agonists of the 5-HT_1D_R with lower potencies than that of 5-HT (1.7 ± 0.3 nM; 100 ± 18%).

The results at the 5-HT_1A_R showed that ibogalogs did not activate this receptor subtype at concentrations as high as 10 µM, especially when compared to that for 5-HT (EC_50_ = 21 ± 6 nM) (Fig. 7B; Table 2). Thus, we assessed whether ibogalogs may act as competitive antagonists against the stimulatory activity of 100 nM 5-HT (Fig. 7C). Ibogalogs at a concentration of 10 μM did not inhibit 5-HT-activated 5-HT_1A_Rs. In comparison, the potent 5-HT_1A_R antagonist WAY100635 [36] showed full inhibition, with an IC_50_ of 0.20 ± 0.07 nM.

The results for the 5-HT_2A_R showed that nor-IBG activated this receptor subtype in the nM concentration range (Fig. 7D; Table 2). Using our previous data on other ibogalogs [16], the following potency sequence was obtained: DM506 (9.0 ± 1.8 nM) > IBG (28 ± 5 nM) > nor-IBG (135 ± 13 nM) > TBG (197 ± 22 nM). Compared to the efficacy of 5-HT (100 ± 1%), TBG (91 ± 18%) and IBG (93 ± 16%) were the most efficient agonists, whereas DM506 (76 ± 16%) and nor-IBG (86 ± 2%) behaved as partial agonists (Table 2). Nor-IBG, at concentrations up to 100 μM, did not activate 5-HT_2B_R but mostly behaved as a competitive antagonist (Fig. 7E; Table 2), which is in agreement with previous results for TBG and IBG [16]. Although the same ibogalogs potently activated the 5-HT_2C_R [16], the potency sequence was different from that of the 5-HT_2A_R: IBG (4.0 ± 1.4 nM) > TBG (13 ± 4 nM) ~ DM506 (33 ± 23 nM) > nor-IBG (52 ± 8 nM) (Fig. 7F; Table 2). Compared to the 5-HT efficacy (100 ± 3%), nor-IBG (98 ± 2%) can be considered a full agonist, as in the case of TBG (99 ± 7%) and IBG (97 ± 8%) [16]. An important structural aspect is that the hydroxyl group in nor-IBG decreases the potency compared to that of IBG, which has a methoxy group in the same position (see Fig. 1), and this difference is more pronounced in the 5-HT_2C_R than in the 5-HT_2A_R. The observed differences among the 5-HT_2A/2C_R subtypes indicated that although ibogalogs are potent agonists, they differ in potency and efficacy.

To assess whether the function of each 5-HT_2_R subtype was modified after prolonged exposure to ibogalog, NIH/3T3 cells containing each 5-HT_2_R subtype were pre-incubated with nor-IBG for 120 h, and the activity of 5-HT was subsequently determined (Fig. 7D–F). One-way ANOVA and Bonferroni’s post hoc test revealed that the potency and efficacy of 5-HT at the 5-HT_2A_R (9.4 ± 0.6 nM; 100 ± 1%) were significantly decreased by 1 μM nor-IBG [16 ± 1 nM (p < 0.005); 93 ± 1% (p < 0.0001)], but not by 0.1 μM nor-IBG (10 ± 1 nM; 100 ± 1%). In the 5-HT_2B_R, the potency and efficacy of 5-HT (16 ± 7 nM; 100 ± 9%) were significantly reduced by 1 μM nor-IBG [165 ± 126 nM (p < 0.05); 52 ± 8% (p < 0.005)], but not by 0.1 μM nor-IBG [16 ± 9 nM (p > 0.9999); 89 ± 10% (p = 0.3526)]. In the 5-HT_2C_R, the potency of 5-HT (0.4 ± 0.1 nM) was modified neither by 0.1 μM (0.6 ± 0.3 nM) (p = 0.9678) nor 1 μM nor-IBG (1.3 ± 0.5 nM) (p = 0.2099), whereas the efficacy of 5-HT (100 ± 3%) was significantly decreased in the presence of 0.1 μM (76 ± 4%) or 1 μM nor-IBG (80 ± 4%) (p < 0.005 for both).

## 4. Discussion

This study focused on assessing the potential of ibogalogs to counteract PTZ-induced seizure activity in mice under acute and repeated treatment paradigms. Furthermore, we studied hippocampal network function through local field potential recordings in hippocampal slices and quantified the tissue concentrations of monoamines and their metabolites in hippocampal tissue.

Behavioral findings indicated that administering acute doses of nor-IBG (30 mg/kg), DM506 (25 or 35 mg/kg), or IBG (10 mg/kg) significantly reduced seizure-like behavior in mice co-injected with PTZ, whereas lower doses did not. Although all compounds started inducing antiseizure effects very rapidly (<10 min post-injection), maximal effects were reached at 20–30 min. However, the scores did not return to the vehicle level, indicating a partial protection. Interestingly, the acute antiseizure activity of ibogalogs was attenuated by volinanserin and SB242084, consistent with an important role for 5-HT_2A/2C_Rs. These findings corroborate those of previous studies, indicating that selective agonists of the 5-HT_2A/2C_R subtypes decrease seizures in rodents, whereas their respective antagonists exacerbate them. Moreover, KO mice exhibit a lower seizure threshold and increased susceptibility to lethality in response to KA administration [2,5,10,11]. Further studies have found that the activation of 5-HT_1A/1D_R, 5-HT_3_R, and 5-HT_4_R subtypes, along with the inhibition of 5-HT_6_R and 5-HT_7_R subtypes, can reduce seizures in rodents [2,5,11,12,37,38]. Ibogalogs showed weak or no stimulatory effects on the 5-HT_1A_R (this study), 5-HT_3_R, 5-HT_4_R, and 5-HT_7_R subtypes [13,16,39]. In contrast, they acted as potent 5-HT_6_R agonists with EC_50_ values in the nM concentration range: DM506 (2.9 ± 0.9 nM), IBG (7.1 ± 2.2 nM), TBG (132 ± 38 nM), and nor-IBG (3.6 ± 3.4 nM). These findings do not support stimulatory activity at 5-HT_1A_R or inhibitory activity at 5-HT_6_R to attain beneficial effects. In addition, ibogalogs activated 5-HT_1D_R with lower potency than that for 5-HT_2A/2C_Rs (this study). More precisely, the potency at the 5-HT_2C_R was 3-(DM506), 8- (nor-IBG), and 165-fold (IBG) higher than that at the 5-HT_1D_R, with a similar trend at 5-HT_2A_R. These data clearly showed that ibogalog-induced 5-HT_2A/2C_R activation is a major mechanism establishing the observed antiseizure activity in mice, whereas the available evidence does not support a role for the other receptor subtypes.

Repeated treatment with a subthreshold dose of nor-IBG (3 mg/kg) or DM506 (5 mg/kg) showed a significant reduction in seizure activity in mice after 7 or 14 days of treatment. Across repeated dosing conditions, both nor-IBG and DM506 produced robust antiseizure effects against PTZ. Although in some time intervals, nor-IBG, but not DM506, reduced PTZ-evoked seizure scores reaching vehicle values, we avoid inferring a single overall efficacy hierarchy between both ibogalogs. The antiseizure activity of ibogalogs elicited after repetitive treatment with subthreshold doses was much stronger than that observed after acute treatment with higher doses. These results indicated that repeated administration maintains antiseizure efficacy without noticeable adverse effects. Although tolerance was not assessed, the sustained effect may suggest that overt tolerance is unlikely under the conditions tested, consistent with a prior report on ibogalogs [16]. Considering that these doses were subthreshold under acute conditions, the emergence of significant effects after repeated administration is consistent with a time-dependent increase in functional impact under the present dosing regimen. The possibility that ibogalogs can accumulate at concentrations high enough to modulate other 5-HTR subtypes can be discarded based on pharmacokinetic results showing that TBG is completely eliminated in approximately 3 h [13]. Thus, we assessed the effect of nor-IBG on 5-HT_2_R desensitization and/or downregulation to test the possibility of a cellular correlation. In this regard, 5-HT_2_R-expressing NIH/3T3 cells were incubated with nor-IBG for 5 days, and the potency of 5-HT was subsequently determined. We found that prolonged incubation with nor-IBG significantly decreased 5-HT potency for the 5-HT_2B_R (10-fold) and 5-HT_2C_R (3-fold), as well as 5-HT efficacy for the 5-HT_2B_R (by 48%) and 5-HT_2C_R (by 24%), with a lesser effect at the 5-HT_2A_R. These results suggest that prolonged ibogalog exposure preferentially induces receptor desensitization rather than downregulation. Our results support previous observations indicating that sustained 5-HT_2A/2C_R activation may decrease binding occupancy (65%) and binding affinity (50%) [40–42]. However, we cannot conclude whether this increased desensitization underlies the enhanced antiseizure efficacy observed after repeated treatments. An alternative mechanism is that ibogalogs, like other psychoplastogens, might stimulate neuritogenesis and synaptogenesis [18,43,44], ameliorating neuronal damage produced during seizure activity [45].

Our experiments monitoring monoamine content in the hippocampus provide additional insights into the effects induced by prolonged exposure to ibogalogs. Acute treatment with ibogalogs did not modify the tissue levels of monoamines or their metabolites per se, or the effect of PTZ on monoamines. This is consistent with the finding that other 5-HT_2C_R and 5-HT_2A_R agonists did not alter the tissue monoamine content in the rat hippocampus [46–49]. However, repeated treatment with subthreshold doses of each ibogalog blunted the effects of PTZ on hippocampal monoamine levels, with a broader attenuation pattern for DM506 across analytes than for nor-IBG. This correlation suggests that DM506-induced 5-HT_2A/2C_R activation is associated with the normalization of PTZ-related monoamine changes (DA, 5-HT, and NE). However, this neurochemical endpoint should not be interpreted as a global indicator of superior antiseizure efficacy for DM506. In this regard, the concomitant activation of DA D2 receptors in the limbic system [50] and/or NE-induced vagus nerve stimulation [51] may contribute to antiseizure effects and warrants further investigation. The inhibitory effect of ibogalogs on monoamine reuptake, particularly that of 5-HT and NE transporters [16], may also play a role.

Previous studies have shown that DM506 decreases GABAergic tone in the prelimbic cortex [52]. However, ibogalogs were found to weakly inhibit potential antiseizure targets such as GABA_A_R and GABA or glycine transporters [13,23]. Conversely, TBG did not increase Glu bursts in the medial prefrontal cortex (mPFC) [18]. This may be related to the weak inhibitory effect of ibogalogs on the Glu transporter or voltage-gated Ca^2+^ channels, both of which modulate synaptic Glu availability [3–5]. Overall, these results do not support a generalized increase or decrease in excitatory transmission as the primary cortical mechanism for the antiseizure activity of ibogalogs [6–9,53]. Perhaps, the antiseizure mechanism is more subtle and involves synaptic rebalancing in specific brain areas different from the cortex, including the hippocampus, amygdala, thalamus, and subthalamic nucleus [6,54–56].

Therefore, we assessed the effects of ibogalogs on KA-treated hippocampal CA3 slices. Nor-IBG reduced KA-induced discharges more efficiently than DM506 at matched concentrations, which is mostly evidenced at longer infusion times. Volinanserin counteracted the effect elicited by nor-IBG on KA-induced epileptiform discharges, supporting the involvement of the 5-HT_2A_R. Since DM506 activates the 5-HT_2A_R, we inferred that DM506-reduced KA-induced discharges is also mediated by the same receptor subtype. Nevertheless, considering that 5-HT_2C_R agonists decrease peak frequency discharge in a polygenic rat model of epilepsy as well as decrease different types of epilepsy [2,5,10,11], we cannot completely discard a potential role for this receptor subtype in the antiseizure activity of ibogalogs, especially because they also act as potent 5-HT_2C_R agonists [16].

Hippocampal CA3 pyramidal cells project indirectly to the CA1 subfield via Schaffer collaterals. In the CA3 region, pyramidal cells (glutamatergic) and interneurons (GABAergic) express 5-HT_2A_Rs [57–59]. Activation of presynaptic CA3 neurons induces Glu release, thereby increasing the firing rates of postsynaptic CA1 neurons via NMDA receptors (NMDARs.) In addition, ibogalogs activate 5-HT_2A_Rs in pyramidal cells [54]. Conversely, 5-HT_2A_R activation in interneurons increases GABA release [59–61]. Therefore, it is conceivable that ibogalog-induced 5-HT_2A_R activation increases inhibitory (GABAergic) transmission, thereby counteracting the excessive excitatory transmission elicited by PTZ or KA in the hippocampus. This interpretation is further supported by recent evidence demonstrating that a closely related receptor subtype, 5-HT_2C_R, enhances GABAergic tone and reduces glutamatergic drive [60]. Moreover, the functional organization of 5-HT_2A_Rs in hippocampal and amygdalo-hippocampal circuits, where they modulate both pyramidal neurons and interneurons, provides an anatomical and physiological basis for these inhibitory shifts [59]. Taken together, these findings indicate that ibogalog-induced 5-HT_2A_R activation may promote a local inhibitory shift in the CA3 region, thereby limiting excessive excitatory activity and contributing to the observed antiseizure effects.

In conclusion, ibogalogs induce antiseizure activity in rodents in a dose- and timeframe-dependent manner, involving 5-HT_2A/2C_R activation. In the hippocampal CA3 subfield, DM506 and nor-IBG suppress KA-induced discharges by a mechanism, clearly demonstrated for nor-IBG, involving 5-HT_2A_R activation. Considering that the antiseizure effects were assessed in young adult mice (6 weeks) and hippocampal slices obtained from young rats (4–6 weeks), extrapolation of these findings to older animals or age-dependent epilepsy contexts should be made with caution. The observed correlation between the beneficial effects of ibogalogs after repeated treatment and concomitant changes in the hippocampal monoamine systems raises additional neurochemical mechanisms. These findings highlight the therapeutic relevance of targeting 5-HT_2A/2C_Rs in modulating network excitability and support further exploration of ibogalogs as candidate antiseizure agents.

## Figures and Tables

**Table 1:** Two-way ANOVA (treatment × time) outputs for Fig. 3 (acute administration) and 4 (repeated administration). Treatment was considered between-subject factor and time bin (0–10, 10–20, 20–30 min) and within-subject repeated- measures factor. Reported values correspond to main effects of treatment and time, and the treatment × time interaction. Post-hoc analyses were performed separately (see figure legends).

Figure/Panel	Effect	F (df1, df2)	p value
Fig. 3A (DM506, acute)	Treatment	F_(6, 189)_ = 937.4	<0.0001
Fig. 3A (DM506, acute)	Time	F_(2, 189)_ = 73.45	<0.0001
Fig. 3A (DM506, acute)	Treatment × Time	F_(12, 189)_ = 2.169	= 0.0147
Fig. 3B (nor-IBG, acute)	Treatment	F_(6, 189)_ = 649.9	<0.0001
Fig. 3B (nor-IBG, acute)	Time	F_(2, 189)_ = 2.027	= 0.1345
Fig. 3B (nor-IBG, acute)	Treatment × Time	F_(12, 189)_ = 8.272	<0.0001
Fig. 4A (DM506, repeated dosing)	Treatment	F_(3, 108)_ = 513.7	<0.0001
Fig. 4A (DM506, repeated dosing)	Time	F_(2, 108)_ = 0.3317	= 0.7184
Fig. 4A (DM506, repeated dosing)	Treatment × Time	F_(6, 108)_ = 0.2685	= 0.9505
Fig. 4B (nor-IBG, repeated dosing)	Treatment	F_(6, 189)_ = 649.9	<0.0001
Fig. 4B (nor-IBG, repeated dosing)	Time	F_(2, 189)_ = 2.027	= 0.1345
Fig. 4B (nor-IBG, repeated dosing)	Treatment × Time	F_(12, 189)_ = 8.272	<0.0001

**Table 2:** Functional activity of ibogalogs at different serotonin receptor subtypes.

Receptor Subtype	Cell type	Compound	EC_50_ (nM)	E_max_ (%)
5-HT_1A_R	HEK293-T	DM506	NA	—
		IBG	NA	—
		Nor-IBG	NA	—
		TBG	NA	—
		CAG	NA	—
		5-HT	21 ± 6	100 ± 5
5-HT_1D_R	HEK293-T	DM506	110 ± 20	89 ± 3
		IBG	661 ± 182	59 ± 12
		Nor-IBG	447 ± 134	75 ± 7
		TBG	209 ± 58	61 ± 7
		CAG	407 ± 164	40 ± 6
		5-HT	1.7 ± 0.3	100 ± 18
5-HT_2A_R	NIH/3T3	Nor-IBG	135 ± 13	86 ± 2
		5-HT	9.4 ± 0.6	100 ± 1
		5-HT (0.1 μM nor-IBG)	10 ± 1	100 ± 1
		5-HT (1 μM nor-IBG)	16 ± 1	93 ± 1
5-HT_2B_R	NIH/3T3	Nor-IBG	>100 μM	—
		5-HT	16 ± 7	100 ± 9
		5-HT (0.1 μM nor-IBG)	16 ± 9	89 ± 10
		5-HT (1 μM nor-IBG)	165 ± 126	52 ± 8
5-HT_2C_R	NIH/3T3	Nor-IBG	52 ± 8	98 ± 2
		5-HT	0.4 ± 0.1	100 ± 3
		5-HT (0.1 μM nor-IBG)	0.6 ± 0.3	76 ± 4
		5-HT (1 μM nor-IBG)	1.3 ± 0.5	80 ± 4

Values are expressed as mean ± SEM.

The EC_50_ and E_max_ values were obtained from Fig. 7.

The E_max_ percentages were calculated considering 100% activation by 5-HT.

NA, no observed activity at 10 μM.

## Data Availability

Data will be made available on request.

## Abbreviation:

5-HT: serotonin
5-HTxR: serotonin receptor subtypes (x = 1A, 1B, 1D, 2A, 2B, 2C, 4, 6, or 7)
GABA: γ-aminobutyric acid
KO: knockout
8-OH-DPAT: 8-hydroxy-2-(di-n-propylamino)tetralin
DM506 (ibogaminalog): 3-methyl-1,2,3,4,5,6-hexahydroazepino[4,5–b]indole
TBG (tabernanthalog): 8-methoxy-3-methyl-1,2,3,4,5,6-hexahydroazepino[4,5–b]indole
IBG (ibogainalog): 9-methoxy-3-methyl-1,2,3,4,5,6-hexahydroazepino[4,5–b]indole
nor-IBG (nor-ibogainalog): 3-methyl-1,2,3,4,5,6-hexahydroazepino[4,5–b]indol-9-ol
IP1: inositol monophosphate 1
BRET: bioluminescence resonance energy transfer
PTZ: pentylenetetrazol
KA: kainic acid
aCSF: artificial cerebrospinal fluid
RT: room temeperature
NE: norepinephrine
DA: dopamine
Glu: glutamate
DOPAC: 3,4-dihydroxyphenylacetic acid
5-HTP: 5-HIAA, 5-hydroxyindole-3-acetic acid
LFPs: local field potentials
ILAE: International League Against Epilepsy
